# Prediction of amphipathic in-plane membrane anchors in monotopic proteins using a SVM classifier

**DOI:** 10.1186/1471-2105-7-255

**Published:** 2006-05-16

**Authors:** Nicolas Sapay, Yann Guermeur, Gilbert Deléage

**Affiliations:** 1 Institut de Biologie et Chimie des Protéines, UMR 5086 CNRS-Univ. Lyon 1 – IFR128 BioSciences Lyon-Gerland, F-69367 Lyon Cedex 07, France; 2 LORIA-CNRS, Campus Scientifique – BP 239, 54506 Vandœuvre-lès-Nancy Cedex, France

## Abstract

**Background:**

Membrane proteins are estimated to represent about 25% of open reading frames in fully sequenced genomes. However, the experimental study of proteins remains difficult. Considerable efforts have thus been made to develop prediction methods. Most of these were conceived to detect transmembrane helices in polytopic proteins. Alternatively, a membrane protein can be monotopic and anchored *via *an amphipathic helix inserted in a parallel way to the membrane interface, so-called in-plane membrane (IPM) anchors. This type of membrane anchor is still poorly understood and no suitable prediction method is currently available.

**Results:**

We report here the "AmphipaSeeK" method developed to predict IPM anchors. It uses a set of 21 reported examples of IPM anchored proteins. The method is based on a pattern recognition Support Vector Machine with a dedicated kernel.

**Conclusion:**

AmphipaSeeK was shown to be highly specific, in contrast with classically used methods (*e.g*. hydrophobic moment). Additionally, it has been able to retrieve IPM anchors in naively tested sets of transmembrane proteins (*e.g*. PagP). AmphipaSeek and the list of the 21 IPM anchored proteins is available on NPS@, our protein sequence analysis server.

## Background

About 25% of open reading frames in fully sequenced genomes are estimated to encode membrane proteins [1]. However, the global analysis of these proteins has proved to be difficult. A greater effort has thus been undertaken to develop prediction methods, with reasonable success [2-4]. Most of these have been devised to detect transmembrane segments with an α-helical conformation (TM helices). This type of membrane segment is the most studied so far, and consequently the most represented in membrane protein databases [5,6]. Alternatively, membrane proteins can be monotopic, *i.e*. bound to the membrane interface and thus in contact with only one of the compartments defined by the membrane. In the latter case, the membrane anchor can be made of (1) covalent links to a hydrophobic compound [7] (2) electrostatic binding to phospholipid head groups [8], (3) hydrophobic loops inserted in the membrane interface [9,10] and (5) amphipathic α-helices inserted at the membrane interface, parallel to the membrane plane, so-called in-plane membrane anchors (IPM anchors) [11,12].

IPM anchors are not uncommon. Since their first discovery in 1986 [13], new examples are regularly reported in the literature. However, IPM anchors are still poorly understood and no suitable prediction method is yet widely available to the scientific community. To date, their analysis *in silico *mainly involves the calculation of the hydrophobic moment [14] and the Schiffer-Edmundson projection [15]. These 2 methods are suitable for depicting amphipathic structures in proteins (*e.g*. [16]), but are not specifically designed for IPM anchors. In fact, they appear as highly sensitive but poorly specific in this latter case. To our knowledge, there has been only one attempt to develop a prediction method for such membrane anchors. It consists of calculating the Depth-Weighted Inserted Hydrophobicity (DWIH, [17]). However, this method has only been assessed on 6 sequences. The main problem springs from the fact that systematic sequence analyses are still limited to a few examples of membrane proteins [17,18]. There is no exhaustive and reliable set of experimentally characterized IPM anchored proteins, making the development of a prediction method very difficult.

In this paper, we describe the first attempt to develop a prediction method for IPM anchors in monotopic proteins using experimental data. In practical terms, our method uses a set of 21 monotopic proteins reported as anchored in the membrane plane. This set constitutes the most exhaustive database of IPM anchored proteins to date. The method is a one-against-all classification process (IPM *versus *non-IPM) based on a pattern recognition Support Vector Machine (SVM) with a dedicated kernel. In contrast with other classically used methods, our objective was to develop a highly specific classifier. Multiple alignments and a hierarchical architecture were additionally used to improve the performances of the SVM. This resulted in an increase of specificity and a limited but significant increase of sensitivity. Our method was naively tested on set of known membrane or soluble proteins, as a key proof of efficiency. It as been able to retrieve IPM segments in several membrane proteins while limiting the prediction of IPM anchors in soluble proteins. Our method, "AmphipaSeeK", was implemented on the NSP@ server [19].

## Results

### Data set building and characterization

**Figure 1:**
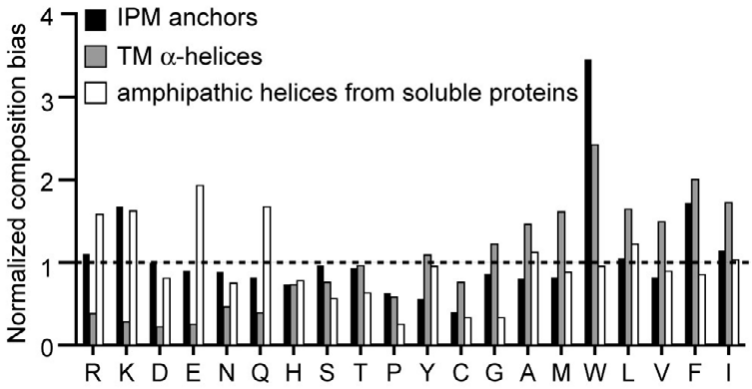
**Amino acid composition bias of IPM anchors, solvent-accessible helices from globular proteins and TM anchors**. Amino acid frequencies were normalized to UniProt amino acid composition (dashed line). The composition of IPM anchors is shown in black, of TM helices in grey and of solvent-accessible helices from globular proteins in white. IPM anchors are extracted from our final data set. Solvent accessible helices are extracted from globular soluble proteins present in the PDB (sequence similarity lower than 25%, accessibility computed by DSSP [53] lower than or equal to 60). TM helices are extracted from the 3D_helix set of the MPtopo database [5].

As detailed in Methods Section, the 21 sequences of monotopic proteins reported as IPM anchored (initial set) were submitted to an enrichment protocol resulting in a homogenous final data set of 91 sequences (enriched set). It is important to note that in this latter set only 7.8% of the residues are involved in an IPM anchor. Their composition bias is reported in Figure 1. The average size of IPM anchors is 23 ± 10 residues and they are mainly predicted in helical and random coil states (66.1% and 28.3% of the residues, respectively). Most of IPM anchors include a single amphipathic α-helix, for a maximum of 3. Finally, IPM anchors appear indifferently located between the extremities or in the middle of the sequences.

In IPM anchors, Lys, Phe and Trp are the most over-represented residues while Cys, Tyr and Pro are the most under-represented. IPM anchors are more hydrophobic than solvent accessible helices from globular proteins, known to be preferentially amphipathic [16,20]. This difference is particularly marked for Trp and Phe, two large hydrophobic residues. As expected, IPM anchors are more hydrophilic than TM helices [16,21]. It is noticeable that Trp is the only hydrophobic residue more abundant in IPM anchors than in TM helices. Trp, Tyr and Lys, are known to be preferentially located at the membrane interface in TM proteins [21,22]. It is then not surprising to observe an over-representation of Trp and Lys in IPM anchors. In contrast, Tyr is under-represented in this type of anchor. However, this fact is difficult to interpret without a larger data set of monotopic proteins.

### Sequence-to-topology SVM: prediction using a single sequence

As the main characteristics of the IPM anchors are an α-helical conformation and a membrane localization, we used the Levin-Robson-Garnier (LRG) [23,24] and PHAT [25] substitution matrices (or more precisely the corresponding Gram matrices) for the SVM Gaussian kernel (see Equations 1 and 2 in Methods section). The LRG matrix was specifically designed for protein secondary structure prediction (e.g. the SOPMA method [26]) while the PHAT matrix is built from predicted TM regions of the Blocks database. The BLOSUM matrix [27] has also been tested but gives a significantly lower performance (data not shown).

The optimal values of the window size, the soft margin parameter *C *and the kernel bandwidth *1/2*σ^2 ^(Equation 1) were determined for each matrix, with and without positional weighting (no positional weighting simply means that the components of the positional weighting vector θ are all set to 1). A ratio of the dual objective function over the primal objective function exceeding 0.90 was used as the stopping criterion for the training procedure. The best results obtained are reported in Table 1. The results obtained with a multi-layer perceptron (MLP) [28,29], a standard connectionist architecture, are also given for comparison. Performance of the SVM trained with the initial set of 21 proteins was measured by using a standard leave-one-out procedure in order to assess the influence of the enrichment protocol. No significant difference has been observed with the SVM trained with the enriched data set (Table 1 in Result section and Table S2 of Additional file 1).

Residues involved in an IPM anchor represent only 7.8% of the total number of residues in the enriched data set. The recognition rate and specificity are consequently not very significant for assessing the quality of the prediction. We have thus used the positive predictive value (P_IMP_), the negative predictive value (P_non-IPM_) and the correlation coefficient of Pearson-Matthews (C_PM_) (Equations 6–8) to better assess the classification performance. Performance with respect to these latter criteria, especially sensitivity, remains low for both matrices when no positional weighting is used.

**Figure 2:**
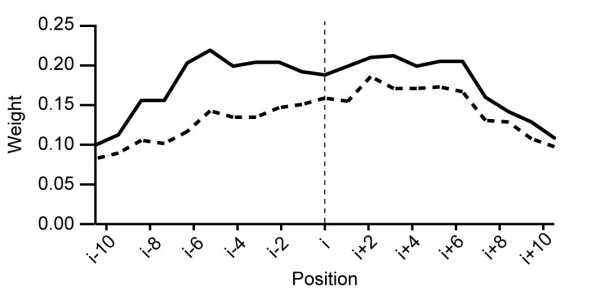
Positional weighting profiles associated with the LRG (dashed line) and PHAT (solid line) matrices.

The introduction of positional weighting dramatically improves prediction accuracy. The profile associated with PHAT (Figure 2), is approximately symmetric with higher weights (> 0.2) at positions *i-6*, *i-5*, *i-3*, *i-2 *and *i+2*, *i+3*, *i+5*, *i+6*, with *i *the absolute position in the sequence of the residue to be classified. The profile associated with LRG is rather asymmetric. Higher weights are found in the right-hand side of the profile.

The results obtained with a positional weighting are similar for both PHAT and LRG. The IPM anchors are largely under-predicted. However, the sensitivity is slightly better with LRG (28.4%) than with PHAT (27.2%). In both cases, predictions are specific with a P_IPM _of 67.0% and 76.3% for LRG and PHAT respectively. The C_PM _is only slightly better when using PHAT. These results call for improvements in the prediction method, in order to improve some measures of accuracy, especially sensitivity. Several options have been investigated, among which we favored two: a hierarchical approach to prediction, with a post-processing of the output, and the introduction of additional evolutionary information.

### Hierarchical approach: topology-to-topology SVM

The output of the sequence-to-topology SVM was used as input of a second SVM, implementing a classical Gaussian kernel. This "topology-to-topology SVM" will be said to be associated with LRG or PHAT, depending on the nature of the substitution matrix used by the sequence-to-topology SVM. Applying such a hierarchical approach to data processing provides us with the possibility of (1) introducing a smoothing to limit aberrant predictions, such as too short IPM segments and (2) taking into account additional pieces of information, for instance the predicted secondary structure. The generalization performance of the topology-to-topology SVM is summarized in Table 2 (values directly comparable to those of Table 1).

The sensitivity of the topology-to-topology SVM is 1.5 times higher than that obtained by the sequence-to-topology SVM using the LRG matrix (42.9% *versus *28.4%, respectively). The sensitivity becomes 2.4 higher when considering the PHAT matrix (64.3% *versus *27.2%, respectively). However P_IPM _is divided by 1.5 for both matrices. The C_PM _is consequently not significantly different between sequence-to-topology and topology-to-topology SVMs when considering the LRG matrix. The performance improvement is more effective with PHAT since the C_PM _is 1.5 times higher than for the corresponding sequence-to-topology SVM. The improvement of the C_PM _is still observed when the predicted secondary structure is included in the input of the topology-to-topology SVM associated to PHAT and is > 0.5. The C_PM _is thus intermediate between those obtained by the sequence-to-topology SVM and by the topology-to-topology SVM without using secondary structures. Additionally, the loss of specificity is less important.

In parallel with the secondary structure, one could wonder whether the hydrophobic moment μH could be used in the input of the topology-to-topology SVM, since μH is commonly calculated to characterize amphipathic helices [14]. In fact, μH quantifies the segregation of hydrophobic and hydrophilic residues along the main axis of an α-helix. However, our preliminary analyses highlighted the fact that high μH values are not specifically associated with IPM anchors (data not shown). Indeed, soluble globular proteins possess numerous amphipathic helices on their surface that do not specifically interact with membranes [16]. Amphipathic helices of IPM anchors are thus completely included in the very abundant population of amphipathic helices from soluble proteins. This is the reason why we have not considered μH.

### Taking into account the evolutionary information using multiple alignments

In order to include additional evolutionary information in our method, we applied the sequence-to-topology SVM to multiple alignments. More precisely, the procedure consists in performing the prediction independently for all the sequences in the alignment, then afterwards deriving a consensus prediction, using a weighted average. This procedure is similar to what was done by [30]. The other standard possibility, to feed the SVM directly with the multiple alignments in place of the sole sequences, would also have been possible (see [31] for details on the way this change affects the computation of the kernel). Since this work is highly time-consuming, this will be done as soon as the parallelization of the M-SVM code will be completed. Aligned sequences for the 91 base sequences were retrieved in UniProt using a previously described process [32]. Different alignment weighting methods were applied for the average score computation: the BLOSUM method [27], a position-based method [33], a Voronoï method [34] and a maximum entropy method [35]. The best results were obtained with the BLOSUM weighting scheme (Table 3, other data not shown).

The performance improvement is significant in both cases. Sensitivity is improved by more than 10%, compared to the sequence-to-topology SVM processing single sequences. The P_IPM _is 1.4 and 1.3 times better for LRG and PHAT respectively. Moreover, the C_PM _exceeds 0.5. This process reduced very efficiently the number of false positives (Tables S1 and S3 of additional data file 1). Since our objective is to build a prediction method as specific as possible, this behavior can be seen as the most satisfactory obtained so far.

### Performance on naively tested sequences

IPM anchors are not the only type of membrane anchors. Furthermore, amphipathic helices are not systematically associated with a membrane. We have thus applied our method to 3 supplementary sets of sequences to test whether it tends to confuse a TM segment or a segment from a soluble protein with an IPM anchor. The first and second sets were composed of membrane proteins of known 3D structure including TM β-barrels or TM helices, respectively. The third set was made up of soluble proteins of known 3D structure that do not interact with a membrane.

Our method was very efficient in distinguishing soluble proteins from membrane proteins since only 57 residues are predicted as "IPM" on a total of 30367 in the set of soluble proteins (0.2% of the residues, see Table 4 and Table S5 of additional file 1). Additionally, more than 80% of the predictions are limited to < 5 consecutive positions. The exception is the β-methylaspartase (PDB: 1KDO) with a predicted IPM segment of 11 residues, corresponding to a solvent-accessible amphipathic helix [36].

Prediction of IPM segments is also limited in TM β-barrel proteins. Only 21 residues on a total of 7699 are predicted as "IPM" in the set of TM β-barrel proteins: 16 of them are involved in a TM β-strand. In this case, predicted IPM anchors are limited to < 3 consecutives residues. Very interestingly, our method predicted an IPM anchor of 6 consecutives residues at the N-terminal extremity of PagP (PDB: 1THQ). This predicted segment indeed corresponds to an amphipathic α-helix perpendicular to the β-barrel and very probably inserted in the membrane plane [37].

The amount and the size of predicted IPM anchors are higher for proteins with TM α-helices: 333 residues on a total of 25813 are predicted as "IPM" (1.3% of the residues). 68% of these predictions have a size > 5 consecutive residues, and 6% a size > 10. Predicted IPM residues are approximately equally distributed between the TM and non-TM parts of the proteins. In fact, most of the predictions of IPM anchors outside a TM helix very likely correspond to effective IPM segments. For example, the 22 C-terminal residues of the subunit L of the photosynthetic reaction center from *Rhodopseudomonas viridis *(PDB: 1DXR) are predicted as "IPM". Analysis of the structure reveals that it indeed corresponds to an amphipathic α-helix perpendicular to a TM α-helix and very likely inserted in the membrane plane (OPM: 1DXR, [38]). Nevertheless, predicted IPM anchors very often overlap the ends of TM α-helices. This problem is not really surprising since the composition biases of the interfacial parts of TM helices and IPM helices appear to be close (Figure 1 and [21]).

Additionally, the 3 sets of proteins were submitted to the SVM trained with the initial set of 21 proteins. Specificity is lower in this case than for the SVM trained with the enriched set (Table S4 of additional file 1). In fact, the SVM trained with the initial set tends to confuse a segment of soluble protein or a TM α-helix with a IPM anchor more often than the SVM trained with the enriched set. In fact, the SVM trained with the initial set tends to be more sensitive and less specific, contrasting with our aim to develop a very specific prediction method.

## Discussion and conclusions

In this paper, we have introduced a prediction method for IPM anchors based on a support vector machine. Our objective was to develop a highly specific classifier in contrast with other methods used to predict this kind of membrane segment (hydrophobic moment, helical wheel projection).

Training was performed using a set of 21 experimentally characterized IPM anchored proteins. The retrieved proteins are involved in various biochemical functions and organisms: viral replication, hormone synthesis in mammals, *etc*.

Our initial set of proteins was enriched using experimental and bioinformatic methods. The final data set contains 91 sequences. This enrichment has allowed us to take into account the important sequence variability between IPM anchors of homologous proteins (e.g. [12] and Brass, Pal *et al*. submitted). The composition bias of the IPM segments shows an over-representation of Lys and Trp, known to be preferentially located at the membrane interface [21,22,39]. Surprisingly, Tyr, also known to be an interfacial residue, is one of the most under-represented residues in IPM anchors. This difference is difficult to interpret because of the limited number of examples reported in the literature. Interestingly, Tyr seems to be preferentially in IPM anchors with low amount of Trp. Thus, Tyr might be also an important membrane determinant, at least for some IPM anchors.

The enriched set was used to train a bi-class SVM, distinguishing the residues involved in an IPM from the other ones. The kernel of this SVM (sequence-to-topology SVM) is a Gaussian function which incorporates an amino acid substitution matrix and a positional weighting vector. Two substitution matrices have been tested: LRG, developed for secondary structure prediction, and PHAT, developed for TM helices prediction. The performance obtained with the 2 matrices is similar: the resulting classifier can be considered as lowly sensitive but specific.

Several possibilities were investigated to improve the prediction accuracy of this classifier. First, its output was used in the input of a second SVM (topology-to-topology SVM), both alone and in conjunction with a prediction of the corresponding secondary structure. This post-processing improves significantly the sensitivity, especially when the sequence-to-topology SVM uses the PHAT matrix. However, the drawback is that the specificity is significantly reduced.

To benefit from the additional evolutionary information in our method, we have used multiple alignments in order to compute average predictions from the sequence-to-topology SVM results. In accordance with our objective, the resulting classifier was very specific. Furthermore, the sensitivity is better than when the prediction is based on the sequences only. Multiple alignments were also used in the two-step approach (sequence-to-topology + topology-to-topology SVM). However, this did not lead to any significant improvement. This is probably due to an overfitting of the topology-to-topology SVM. The implementation of a stacked generalization procedure [40] appears as the natural solution to this problem. This will be done after the completion of the SVM parallelization.

Given the experimental results summarized above, the configuration we eventually selected for our prediction method consists of a sequence-to-topology SVM processing multiple alignments. In accordance with our objective, the method is highly specific (99.8%), with a C_PM _of 0.53. The low sensitivity is difficult to overcome since it is, at least partially, due to the imbalance between the amounts of IPM (7.8%) and non-IPM (92.2%) residues. The imbalance could be influenced by Trp, a residue over-represented in the data set and associated with high scores in substitution matrices. Trp is thus associated with low values in the matrix of dot products between amino acids. Consequently, the classifier could underestimate the "IPM" category in Trp poor sequences.

Unfortunately, our classification method cannot be compared readily with the only other prediction method of IPM anchors published so far, the DWIH measurement (see introduction), for two main reasons. First, the DWIH algorithm is not publicly available; second, its reported efficiency has been measured on 6 sequences only. However, our method has been naively tested on 3 sets of proteins made up of soluble proteins, proteins with TM β-barrels or proteins with TM α-helices. The prediction of IPM anchors is limited in soluble proteins and proteins with TM β-barrels, as expected. In the case of membrane proteins with TM helices, predicted IPM anchors tend to overlap the ends of the TM segments. This is very probably due to the composition bias of these parts of TM helices, rather close to the one of IPM anchors (Figure 1 and [21]). In fact, defining the limit between a TM and an IPM segment in transmembrane proteins is not a trivial problem, even when a 3D structure is available. Including TM proteins in the training set will probably partially solve the problem. However, this will require the systematic annotation of the TM and IPM segments in transmembrane proteins, a long and difficult task. As preliminary tests, we included some well-defined cases of transmembrane proteins with IPM anchors in the training set (*e.g*. gp41 [41]), which gave satisfactory results.

As a final proof of efficacy, our method has been able to retrieve several IPM anchors in transmembrane proteins (*e.g*. PDB: 1THQ). In fact, it would be interesting to turn to a multi-class problem by introducing additional categories, *e.g*. a "TM" category. Note that this would not generate any technical problem since our SVM software is actually a multi-class one. Additionally, it will be interesting to further investigate the choice of the kernel; for example, it is possible to combine several kernels (one dedicated to the sequence, one dedicated to the secondary structure, *etc*.) into a single one (*e.g*. [42]) and to adapt the Gaussian kernel to directly deal with multiple alignments. In any case, a regular update of the initial data set used in our method will improve the performance. Finally, our method, "AmphipaSeeK", is available on the NPS@, our protein sequence analysis server [19].

## Methods

### Data Set

**Figure 3:**
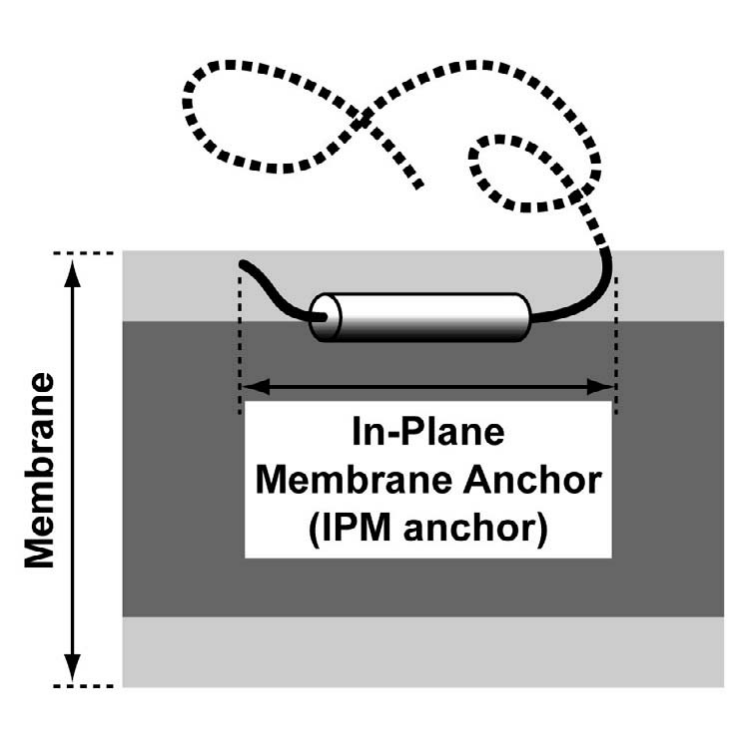
**Schematic representation to scale of an IPM anchor**. The amphipathic α-helix of the IPM anchor is depicted as a black and white cylinder, for the hydrophobic and hydrophilic sides, respectively. The non-membrane part of the protein is represented by a dotted line. The membrane hydrophobic core, including acyl chains, is dark grey and the membrane interface, including glycerol and above atoms, is light grey.

The sequences constituting the experimentally characterized data set were initially retrieved from the literature. The 21 selected sequences correspond to monotopic proteins with an experimentally characterized IPM anchor segment (Figure 3). Experiments included insertion-deletion-mutation, fusion with soluble heterologous proteins (*e.g*. Green Fluorescent Protein), liposome and/or unilamellar vesicule binding assays, and structural studies using circular dichroism, ATR-FTIR and liquid/solid NMR in membrane mimetic media. All sequences possess an unambiguous IPM anchor. This means that the segment including the IPM anchor: (1) is necessary and sufficient for the membrane anchor; (3) is < 75 residues long and is mainly arranged as an α-helix; (4) possesses amphipathic α-helices (characterized or predicted) and (5), no TM anchor is present in the whole protein.

The database was completed with our experimental study of the NS5A N-terminal segment from Hepatitis C Virus (HCV) and related viruses. HCV belongs to the *Flaviviridae *family including *Flavivirus *(*e.g*. dengue virus), *Pestivirus *(*e.g*. bovine viral diarrhea virus or BVDV), *Hepacivirus *(HCV) genera and unclassified GB viruses (GB virus A, B and C). NS5A N-terminal segments of HCV [43], GB viruses B and C (Brass, Pal *et al*., submitted), and BVDV [12] has been demonstrated to be necessary and sufficient to anchor Green Fluorescent Protein to the endoplasmic reticulum membrane. ATR-FTIR experiments have shown that these peptides are positioned parallel to the membrane (Vigano and Huet-Pêcheur, personal communication). Determination of the three-dimensional structure of the membrane segments of the BVDV segment by NMR performed in various membrane mimetic environments has revealed the presence of an amphipathic α-helix positioned at the interface of peptide-detergent micelles [12]. All these experimentally characterized proteins have been included in the data set.

**Figure 4:**
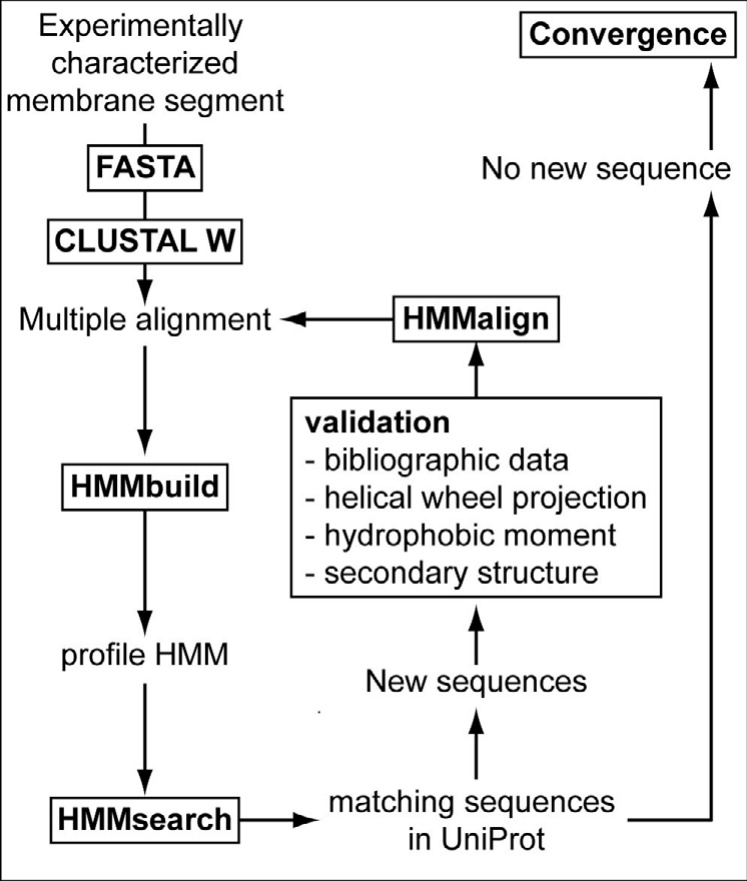
Flowchart of the data set enrichment process.

This initial data set was enriched by the application of a sequence of treatments *in silico *centered on a profile HMM method (Figure 4). The aim was to increase the evolutionary information content of the data set by including distant homologous sequences, since IPM anchors of closely related proteins can have a low sequence similarity (*e.g*. NS5A proteins from HCV, GB viruses and BVDV, [12] and Brass, Pal *et al*. submitted). Thus, this set is considered as enriched since it contains more different examples of IPM anchors, even if the entire protein sequences are globally similar. It must be borne in mind that the enrichment process does not constitute a prediction method itself. Indeed, it tells us nothing about IPM anchors possibly existing in sequences not homologous to those of the data set of reference.

Each experimentally characterized IPM segment was submitted to the FASTA homology search program [44]. Retrieved sequence segments were aligned using CLUSTAL W [45]. HMM profiles were built from these multiple alignments using HMMbuild from the HMMER 2.2 g package [46]. Each profile was searched for in the UniProt database [47] using HMMsearch from the HMMER 2.2 g package. Matching sequence segments extracted from HMMsearch results were evaluated as putative members of the family of IPM anchored proteins by examining (1) the presence of a predicted α-helix with a consensus secondary structure prediction method, the amphiphilicity of predicted helices with (2) helical wheel projections and (3) hydrophobic moment calculation, and (4) by searching for the membrane binding properties of the corresponding sequences reported in the literature, when available. The validated new segments were included in the set of aligned sequences with HMMalign. A new HMM profile was constructed and searched for once more in UniProt. This iterative process was repeated until convergence, *i.e*. when no new segment could be validated and added to the previous multiple alignments. All the above tools are available on the NPS@ Web server [19]. The predicted secondary structure was obtained as a consensus from several prediction methods also available on the NPS@ Web server: DSC, PHD and SOPMA (see NPS@ home page and references therein). Hydrophobic moments of predicted α-helices [14] have been computed using a size 11 sliding window and an angle of 100 deg.

The enrichment process could retrieve 531 sequences comprising some distant homologous sequences and also many closely homologous ones, which contained less useful evolutionary information. To overcome this problem, only IPM segments with a similarity < 50% were selected from the enriched data set, representing 91 sequences. Finally, the full-length sequences corresponding to those 91 segments have been retrieved. They constituted our data set. Their similarity was approximately < 50% but the exact value was not so important since (1) the classification method was a bi-class one (*i.e*. IPM position or not) and (2) the SVM, due to the geometrical nature of the principle on which it is based (maximal margin hyperplane), could deal with redundant information.

### SVM classifier

We have seen earlier that homology could not be used as a single criterion to perform the prediction. The classification method we used was a SVM [48,49]. To overcome the aforementioned shortcomings, it implements a totally difference strategy: the inference of statistical regularities from local information (the content of a sliding window). The conjecture is that the local context tells us something about the state of a residue, precisely if it belongs to an IPM anchor or not, and that this knowledge can be extracted even from non homologous sequences. In that context, the aim of the enrichment process is primarily to provide the classifier with additional information regarding the natural variability it must cope with.

The training algorithm implemented, described in detail in [50], was inspired from the Frank-Wolfe algorithm [51]. The main advantage of this algorithm, which incorporates a decomposition method, consists in making it possible to process very large data sets.

### Choice of the kernel

The predictors used to determine the category of each residue are the amino acids contained in a sliding window centered on the residue to be classified. The description of each example is thus a vector ***x ***= (*x*_*i*_)_-*n*≤*i*≤*n *_of {1,..., 22}^2n+1^, the integers 1 to 20 corresponding to the 20 amino acids, while 21 is used to designate undetermined amino acids (*i.e*. X, B and Z) and 22 corresponds to an empty position (which occurs when the window overlaps with the N or the C-terminus of a sequence). The kernel used by the SVM is the Gaussian kernel introduced in [31]. Compared to the basic implementation of the Gaussian kernel for sequence processing, this one exhibits two specificities: it makes use of a matrix ***D ***= (*d*_*ij*_)_*1*≤*ij*≤*22 *_of dot products between amino acids and a positional weighting vector **θ **= (θ_*i*_)_-*n*≤*i*≤*n*_. It is given by the formula:

kθ,D(x,x′)=exp⁡[∑i=−nnθi2⋅‖xi−x′i‖22σ2]     (1)
 MathType@MTEF@5@5@+=feaafiart1ev1aaatCvAUfKttLearuWrP9MDH5MBPbIqV92AaeXatLxBI9gBaebbnrfifHhDYfgasaacH8akY=wiFfYdH8Gipec8Eeeu0xXdbba9frFj0=OqFfea0dXdd9vqai=hGuQ8kuc9pgc9s8qqaq=dirpe0xb9q8qiLsFr0=vr0=vr0dc8meaabaqaciaacaGaaeqabaqabeGadaaakeaacqWGRbWAdaWgaaWcbaaccmGae8hUdeNaeiilaWccbmGae4hraqeabeaakiabcIcaOiab+Hha4jabcYcaSiqb+Hha4zaafaGaeiykaKIaeyypa0JagiyzauMaeiiEaGNaeiiCaa3aamWaaeaadaWcaaqaamaaqahabaacciGae0hUde3aa0baaSqaaiabdMgaPbqaaiabikdaYaaaaeaacqWGPbqAcqGH9aqpcqGHsislcqWGUbGBaeaacqWGUbGBa0GaeyyeIuoakiabgwSixpaafmaabaGaemiEaG3aaSbaaSqaaiabdMgaPbqabaGccqGHsislcuWG4baEgaqbamaaBaaaleaacqWGPbqAaeqaaaGccaGLjWUaayPcSdWaaWbaaSqabeaacqaIYaGmaaaakeaacqaIYaGmcqqFdpWCdaahaaWcbeqaaiabikdaYaaaaaaakiaawUfacaGLDbaacaWLjaGaaCzcamaabmaabaGaeGymaedacaGLOaGaayzkaaaaaa@6082@

Under the assumption that the amino acids in the *i*-th position of the first and second window are those of indices *j *and *k *(no matter in which order), ||*x*_*i *_- *x*'_*i*_||^2 ^is given by:

||*x*_*i *_- *x*'_*i*_||^2 ^= *d*_*jj *_+ *d*_*kk *_- 2*d*_*jk *_    (2)

Thanks to the use of ***D***, the amino acids (and the unknown amino acids and the empty position) are not supposed to form an orthonormal basis. In other words, the distance between the contents of two positions with equal indices in two windows is not simply 0 (identical contents) or 1 (different contents), but can take different values as a function of the amino acids involved. The components of matrix ***D ***are derived from similarity/substitution matrices. In that way, evolutionary information can be taken into account. The weighting vector **θ **modulates the influence of the different positions in the window on the prediction. Details on the determination of ***D ***and **θ **are given in the following subsection.

### Setting the parameters of the Gaussian kernel

#### Computation of the matrix of dot products *D*

As explained above, the kernel integrates evolutionary information through a matrix of dot products between amino acids. This matrix is directly derived from a substitution matrix. Such matrices cannot be used directly in the computation of the kernel since they are not symmetric positive (semi-)definite, *i.e*. are not associated with an underlying dot product. However, since they are symmetric anyway, one simple way to approximate them with a Gram matrix consists in diagonalizing them and replacing all the negative eigenvalues with 0. This is what was done with the two substitution matrices used in the experiments reported in Results section, LRG and PHAT.

#### Positional weighting vector θ

The determination of the values of the components of vector **θ **in Equation 1 is the result of a supervised learning algorithm. The matrix ***D ***being given, a training set is used to implement a kernel alignment principle introduced in [52]. In short, the objective function with respect to which vector **θ **is optimized is the "fit" between the computed Gram matrix and an ideal one (for which building a classifier with optimal recognition rate and large margin would be trivial). In practice, **θ **is obtained through a stochastic gradient ascent.

### Validation protocol

The procedure implemented to derive the test performance is a standard seven-fold cross-validation. During the procedure, a great care has been taken to put homologous sequences in the same cross-validation subset. Two homologous sequences were then learnt/tested concomitantly. Six different measures were used to assess the prediction accuracy, involving the 4 components of the confusion matrix (TP, number of correctly classified IPM positions; TN, number of correctly classified non-IPM positions; FP, number of incorrectly classified non-IPM positions; FN, number of incorrectly classified IPM positions):

Accuracy:

Acc=TP+TNTP+TN+FP+FN     (3)
 MathType@MTEF@5@5@+=feaafiart1ev1aaatCvAUfKttLearuWrP9MDH5MBPbIqV92AaeXatLxBI9gBaebbnrfifHhDYfgasaacH8akY=wiFfYdH8Gipec8Eeeu0xXdbba9frFj0=OqFfea0dXdd9vqai=hGuQ8kuc9pgc9s8qqaq=dirpe0xb9q8qiLsFr0=vr0=vr0dc8meaabaqaciaacaGaaeqabaqabeGadaaakeaacqqGbbqqcqqGJbWycqqGJbWycqGH9aqpdaWcaaqaaiabbsfaujabbcfaqjabgUcaRiabbsfaujabb6eaobqaaiabbsfaujabbcfaqjabgUcaRiabbsfaujabb6eaojabgUcaRiabbAeagjabbcfaqjabgUcaRiabbAeagjabb6eaobaacaWLjaGaaCzcamaabmaabaGaeG4mamdacaGLOaGaayzkaaaaaa@466E@

Sensitivity:

Sn=TPTP+FN     (4)
 MathType@MTEF@5@5@+=feaafiart1ev1aaatCvAUfKttLearuWrP9MDH5MBPbIqV92AaeXatLxBI9gBaebbnrfifHhDYfgasaacH8akY=wiFfYdH8Gipec8Eeeu0xXdbba9frFj0=OqFfea0dXdd9vqai=hGuQ8kuc9pgc9s8qqaq=dirpe0xb9q8qiLsFr0=vr0=vr0dc8meaabaqaciaacaGaaeqabaqabeGadaaakeaacqqGtbWucqqGUbGBcqGH9aqpdaWcaaqaaiabbsfaujabbcfaqbqaaiabbsfaujabbcfaqjabgUcaRiabbAeagjabb6eaobaacaWLjaGaaCzcamaabmaabaGaeGinaqdacaGLOaGaayzkaaaaaa@3BD9@

Specificity:

Sp=TNTN+FP     (5)
 MathType@MTEF@5@5@+=feaafiart1ev1aaatCvAUfKttLearuWrP9MDH5MBPbIqV92AaeXatLxBI9gBaebbnrfifHhDYfgasaacH8akY=wiFfYdH8Gipec8Eeeu0xXdbba9frFj0=OqFfea0dXdd9vqai=hGuQ8kuc9pgc9s8qqaq=dirpe0xb9q8qiLsFr0=vr0=vr0dc8meaabaqaciaacaGaaeqabaqabeGadaaakeaacqqGtbWucqqGWbaCcqGH9aqpdaWcaaqaaiabbsfaujabb6eaobqaaiabbsfaujabb6eaojabgUcaRiabbAeagjabbcfaqbaacaWLjaGaaCzcamaabmaabaGaeGynaudacaGLOaGaayzkaaaaaa@3BDB@

Positive predictive value, *i.e*. proportion of correctly predicted IPM residues:

PIPM=TPTP+FP     (6)
 MathType@MTEF@5@5@+=feaafiart1ev1aaatCvAUfKttLearuWrP9MDH5MBPbIqV92AaeXatLxBI9gBaebbnrfifHhDYfgasaacH8akY=wiFfYdH8Gipec8Eeeu0xXdbba9frFj0=OqFfea0dXdd9vqai=hGuQ8kuc9pgc9s8qqaq=dirpe0xb9q8qiLsFr0=vr0=vr0dc8meaabaqaciaacaGaaeqabaqabeGadaaakeaacqqGqbaudaWgaaWcbaGaeeysaKKaeeiuaaLaeeyta0eabeaakiabg2da9maalaaabaGaeeivaqLaeeiuaafabaGaeeivaqLaeeiuaaLaey4kaSIaeeOrayKaeeiuaafaaiaaxMaacaWLjaWaaeWaaeaacqaI2aGnaiaawIcacaGLPaaaaaa@3E0F@

Negative predictive value, *i.e*. proportion of correctly predicted non-IPM residues:

Pnon-IPM=TNTN+FN     (7)
 MathType@MTEF@5@5@+=feaafiart1ev1aaatCvAUfKttLearuWrP9MDH5MBPbIqV92AaeXatLxBI9gBaebbnrfifHhDYfgasaacH8akY=wiFfYdH8Gipec8Eeeu0xXdbba9frFj0=OqFfea0dXdd9vqai=hGuQ8kuc9pgc9s8qqaq=dirpe0xb9q8qiLsFr0=vr0=vr0dc8meaabaqaciaacaGaaeqabaqabeGadaaakeaacqqGqbaudaWgaaWcbaGaeeOBa4Maee4Ba8MaeeOBa4Maeeyla0IaeeysaKKaeeiuaaLaeeyta0eabeaakiabg2da9maalaaabaGaeeivaqLaeeOta4eabaGaeeivaqLaeeOta4Kaey4kaSIaeeOrayKaeeOta4eaaiaaxMaacaWLjaWaaeWaaeaacqaI3aWnaiaawIcacaGLPaaaaaa@4311@

Correlation coefficient of Pearson-Matthews:

CPM=TP×TN−FP×FN(TP+FP)(TP+FN)(TN+FP)(TN+FN)     (8)
 MathType@MTEF@5@5@+=feaafiart1ev1aaatCvAUfKttLearuWrP9MDH5MBPbIqV92AaeXatLxBI9gBaebbnrfifHhDYfgasaacH8akY=wiFfYdH8Gipec8Eeeu0xXdbba9frFj0=OqFfea0dXdd9vqai=hGuQ8kuc9pgc9s8qqaq=dirpe0xb9q8qiLsFr0=vr0=vr0dc8meaabaqaciaacaGaaeqabaqabeGadaaakeaacqqGdbWqdaWgaaWcbaGaeeiuaaLaeeyta0eabeaakiabg2da9maalaaabaGaeeivaqLaeeiuaaLaey41aqRaeeivaqLaeeOta4KaeyOeI0IaeeOrayKaeeiuaaLaey41aqRaeeOrayKaeeOta4eabaWaaOaaaeaacqGGOaakcqqGubavcqqGqbaucqGHRaWkcqqGgbGrcqqGqbaucqGGPaqkcqGGOaakcqqGubavcqqGqbaucqGHRaWkcqqGgbGrcqqGobGtcqGGPaqkcqGGOaakcqqGubavcqqGobGtcqGHRaWkcqqGgbGrcqqGqbaucqGGPaqkcqGGOaakcqqGubavcqqGobGtcqGHRaWkcqqGgbGrcqqGobGtcqGGPaqkaSqabaaaaOGaaCzcaiaaxMaadaqadaqaaiabiIda4aGaayjkaiaawMcaaaaa@5FF0@

## Availability and requirements

**Name: **AmphipaSeeK

**Operating system: **platform independent

**Programming language: **C and Python

**Other requirements: **Python 2.4 or higher

**Availability: **AmphipaSeeK is available on the NSP@ server  at the following URL: 

The list of the 21 proteins used to build the data set is available on the AmphipaSeeK help page:



The M-SVM source code is available on the Web page of Yann Guermeur



## Authors' contributions

Nicolas Sapay, Yann Guermeur and Gilbert Deléage planned the project, Yann Guermeur and Nicolas Sapay wrote the method, Nicolas Sapay built the data set of monotopic proteins. All authors wrote the article and approved the final manuscript.

## Supplementary Material

Additional File 1 One file containing Tables S1 to S4. Click here for file

## Figures and Tables

**Table 1:** Sequence-to-topology SVM performance using the LRG and PHAT matrices

	*No Positional Weighting*		*Positional Weighting*		
Substitution Matrix	LRG^a^	PHAT^b^	LRG^c^	PHAT^d^	MLP^e^

Accuracy	94.0	93.6	93.9	94.3	90.6
Sensitivity	18.3	9.9	28.4	27.2	35.3
Specificity	99.8	100.0	98.9	99.4	94.8
P_non-IPM_	94.1	93.6	94.8	94.7	95.1
P_IPM_	87.1	94.4	67.0	76.3	34.2
C_PM_	0.38	0.30	0.41	0.44	0.30

^a ^*C *= 5.0, *1/2*σ^2 ^= 0.03, window size = 21

^b ^*C *= 5.0, *1/2*σ^2 ^= 0.01, window size = 21

^c ^*C *= 25.0, *1/2*σ^2 ^= 0.40, window size = 21 residues

^d ^*C *= 5.0, *1/2*σ^2 ^= 0.10, window size = 21 residues

^e ^hidden layer size = 16, window size = 15 residues

**Table 2:** Topology-to-topology SVM training and test performance using as input the output of the sequence-to-topology SVM. "With" and "Without Structure II" indicates if the predicted secondary structure of the sequence is also included in input or not. LRG and PHAT columns correspond to the substitution matrices used by the sequence-to-topology SVM.

	*Without Structure II*		*With Structure II*	
Substitution Matrix	LRG^a^	PHAT^b^	LRG^c^	PHAT^d^

Accuracy	91.7	93.6	92.3	94.3
Sensitivity	42.9	64.3	41.1	44.1
Specificity	95.5	95.9	96.4	98.3
P_non-IPM_	95.4	97.1	95.3	95.6
P_IPM_	44.1	55.7	47.5	67.1
C_PM_	0.39	0.64	0.40	0.52

^a ^*C *= 10.0, *1/2*σ^2 ^= 0.1, predictors = segment of 21 residues

^b ^*C *= 10.0, *1/2*σ^2 ^= 0.1, predictors = segment of 21 residues

^c ^*C *= 15.0, *1/2*σ^2 ^= 0.1, predictors = segment of 21 residues + corresponding predicted secondary structure

^d ^*C *= 5.0, *1/2*σ^2 ^= 0.05, predictors = segment of 21 residues + corresponding predicted secondary structure.

**Table 3:** Quality of the predictions involving multiple alignments. The weights assigned to the aligned sequences are calculated using a BLOSUM weight scheme at a fractional identity of 0.80. LRG and PHAT columns correspond to the substitution matrices used by the SVM.

Substitution Matrix	LRG	PHAT
Accuracy	95.0	95.0
Sensitivity	31.3	31.3
Specificity	99.8	99.8
P_non-IPM_	92.3	93.8
P_IPM_	95.0	95.0
C_PM_	0.52	0.53

**Table 4:** Classification performance for 3 sets of soluble or transmembrane proteins naively tested. "Observed as" corresponds to the number of residues observed at a TM or a non-TM position. "Predicted as" corresponds to the number of residues predicted at a IPM or non-IPM position. "Proteins with TM α-helix" is a set of 101 proteins with 1 or more TM α-helices. "Proteins with TM β-barrel" is a set of 21 TM β-barrel proteins. TM proteins are extracted from the MPtopo database (3D_helix and 3D_other subsets, respectively). "Soluble proteins" is a set of 65 soluble proteins extracted from the PDB (sequence similarity < 25%). These 3 sets were submitted to the sequence-to-topology SVM, using PHAT and a positional weighting (Table 1). An average prediction was then computed for each sequence of the sets following the procedure described above (Table 3).

	**Proteins with TM α-helix**		**Proteins with TM β-barrel**		**Soluble proteins**	
	*Observed as*		*Observed as*		*Observed as*	

*Predicted as*	TM	non-TM	TM	non-TM	TM	non-TM
IPM	181	152	16	5	-	57
non-IPM	11057	14423	3540	4138	-	30310
*Total number of residues*	*11238*	*14575*	*3556*	*4143*	*-*	*30367*
