# Supplementary material for: Prediction of amphipathic in-plane membrane anchors in monotopic proteins using a SVM classifier
Source: BMC Bioinformatics. 2006 May 16;7:255. doi: 10.1186/1471-2105-7-255 (PMC1564421; doi:10.1186/1471-2105-7-255)
Supplement: Additional File 1 — One file containing Tables S1 to S4. [file 1471-2105-7-255-S1.PDF]

Table S4. Performance of the sequence-to-topology SVM (confusion matrix)

Consensus prediction computed on multiple alignments. Average scores were computed using a BLOSUM weight scheme at a fractional identity of 0.80.

Substitution Matrix	LRG	PHAT
True Positives	844	844
True Negatives	35402	35416
False Positives	70	56
False Negatives	1857	1857
Total	*38173*	*38173*

Table S5. Classification performance for 3 sets of soluble or transmembrane proteins naively tested with the initial set of 21 sequences used as training set.

"Observed as" corresponds to the number of residues observed at a TM or a non-TM position. "Predicted as" corresponds to the number of residues predicted at a IPM or non-IPM position. "Proteins with TM α-helix" is a set of 101 proteins with 1 or more TM α-helices. "Proteins with TM β-barrels" is a set of 21 TM β-barrel proteins. TM proteins are extracted from the MPtopo database (3D_helix and 3D_other subsets, respectively). "Soluble proteins" is a set of 65 soluble proteins extracted from the PDB (sequence similarity < 25 %). These 3 sets were submitted to the sequence-to-topology SVM, using PHAT and a positional weighting (Table 1). An average prediction was then computed for each sequence of the sets following the procedure described above (Table 3).

	**Proteins with TM α-helix**		**Proteins with TM β-barrel**		**Soluble proteins**	
	Observed as		*Observed as*		*Observed as*	
Predicted as	TM	non-TM	TM	non-TM	TM	non-TM
IPM	255	317	11	8	-	99
non-IPM	10983	14258	3545	4135	-	30268
Total number of residues	*11238*	*14575*	*3556*	*4143*	-	*30367*

Additional data

Table S1. Training performance of the sequence-to-topology SVM (confusion matrix)

The SVM is used with single sequences and LRG or PHAT substitution matrices. No positional weighting corresponds to a θ vector set to 12n+1. The MLP column corresponds to the performance obtained with a Multi-Layer Perceptron (MLP).

	No Positional Weighting		*Positional Weighting*		
Substitution Matrix	LRG	PHAT	LRG	PHAT	MLP
True Positives	493	268	768	736	954
True Negatives	35399	35456	35093	35243	33635
False Positives	73	16	379	229	1837
False Negatives	2208	2433	1933	1965	1747
Total	*38173*	*38173*	*38173*	*38173*	*38173*

Table S2. Training performance of the sequence-to-topology SVM trained with the initial set of 21 sequences (confusion matrix)

The SVM is used with single sequences and LRG or PHAT substitution matrices. Performances were measured using a standard leave-one-out procedure. A positional weighting has been used.

	No Positional Weighting		*Positional Weighting*	
Substitution Matrix	LRG[a]	PHAT[b]	LRG[c]	PHAT[d]
Accuracy	93.2	93.2	94.1	94.2
Sensitivity	6.1	6.2	26.3	25.1
Specificity	100.0	100.0	99.4	99.6
$P_{non\text{-}IPM}$	93.2	93.2	94.6	94.5
P_{IPM}	100.0	91.3	76.3	81.6
C_{PM}	0.24	0.23	0.43	0.43

[a] C = 5.0, $1/2\sigma^2$ = 0.04, window size = 21
[b] C = 5.0, $1/2\sigma^2$ = 0.0075, window size = 21
[c] C = 5.0, $1/2\sigma^2$ = 0.2, window size = 21
[d] C = 5.0, $1/2\sigma^2$ = 0.2, window size = 21

Table S3. Training performance of the topology-to-topology SVM (confusion matrix)

Input vectors are built using sequence-to-topology SVM scores for each position in the sliding window, with or without their associated predicted secondary structure.

	Without structure II		*With structure II*	
Substitution Matrix	LRG	PHAT	LRG	PHAT
True Positives	1212	1818	1163	1247
True Negatives	33809	33899	34059	34735
False Positives	1537	1447	1287	611
False Negatives	1615	1009	1664	1580
Total	*38173*	*38173*	*38173*	*38173*
